# Management of Tiny Meningiomas: To Resect or Not Resect

**DOI:** 10.7759/cureus.1514

**Published:** 2017-07-25

**Authors:** Julia R Schneider, Kay O Kulason, Tim White, Bidyut Pramanik, Shamik Chakraborty, Linda Heier, Ashley E Ray, Todd A Anderson, Derek J Chong, John Boockvar

**Affiliations:** 1 Neurosurgery, Brain Tumor Center, Lenox Hill Hospital, Hofstra Northwell School of Medicine; 2 Department of Neurosurgery, Hofstra Northwell School of Medicine; 3 Radiology, Lenox Hill Hospital, Hofstra Northwell School of Medicine; 4 Radiology, Weill Cornell Medicine & New York Presbyterian Hospital, New York; 5 Pathology, Lenox Hill Hospital, Hofstra Northwell School of Medicine; 6 Neurology, Lenox Hill Hospital, Hofstra Northwell School of Medicine

**Keywords:** meningioma, management, progesterone receptors, calcification

## Abstract

Meningiomas are most often benign primary intracranial tumors that are frequently found incidentally on imaging. Larger sized meningiomas may present with symptoms such as seizures and headaches. Smaller meningiomas are commonly asymptomatic and usually observed with serial imaging. We present two female patients, both of whom were found to have very small left frontal meningiomas that marginated Broca’s area. The first patient in this case series experienced episodes resembling seizures which consisted of weakness, vision loss, and slurred speech, as well as subtle language dysfunction in her day-to-day conversations. The second patient presented with headaches and an enlarging meningioma. Both meningiomas were surgically resected and the patients’ symptoms resolved. Small meningiomas should not be overlooked as they may very well be the source of neurologic symptoms.

## Introduction

Meningiomas are primary central nervous system (CNS) tumors that arise from arachnoidal cells of the leptomeninges [1]. With an incidence of six to eight cases per 100,000 persons per year, meningiomas are typically benign and slow growing [2-3]. These primary CNS tumors are the most frequently reported tumor, accounting for 36.4% of all brain tumors [2]. The risk of being diagnosed with a meningioma increases with age, especially in individuals 45 years or older [1, 4]. Meningiomas are reported to occur over 2.5 times more frequently in females than in males [1-2]. Often, these tumors are found incidentally on magnetic resonance imaging (MRI) without any presenting symptoms [4]. In patients with symptomatic meningiomas, seizures and headaches are the most common presentations [5]. Surgical resection is warranted to prevent progression and mass effect for large symptomatic tumors that compress surrounding neurological structures [6]. However, the treatment plan for small meningiomas is not so well defined [7]. If calcification is present in a small meningioma, this signifies slow or absent tumor growth prompting the need to closely monitor the lesion without immediate surgical intervention [6-7]. Here, we present two cases in which both of the small meningiomas are located in the left frontal lobe adjacent to Broca’s area. In the first case, the meningioma was calcified and electroencephalogram (EEG) done twice showed no epileptiform activity. In the second case, the meningioma was growing slowly. We describe the management of these two cases with similarly located and sized meningiomas. 

## Case presentation

Case 1

**Figure 1:**
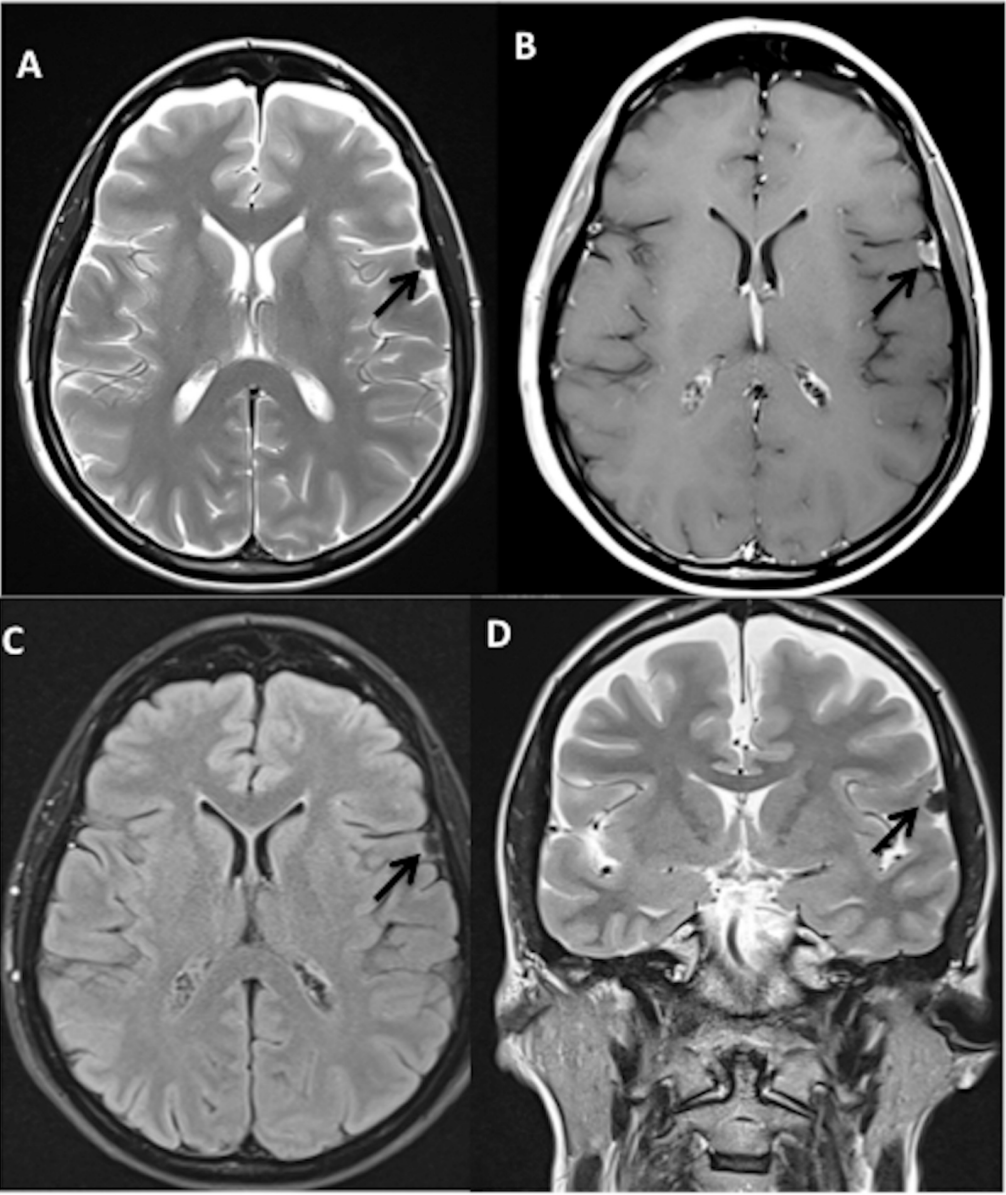
Preoperative magnetic resonance imaging of the brain showing left frontal meningioma (patient 1) Black arrows point to the lesion. A) Axial T2, turbo spin echo, B) Axial T1 post contrast, C) Axial T2, fluid-attenuated inversion recovery, D) Coronal T2

**Figure 2:**
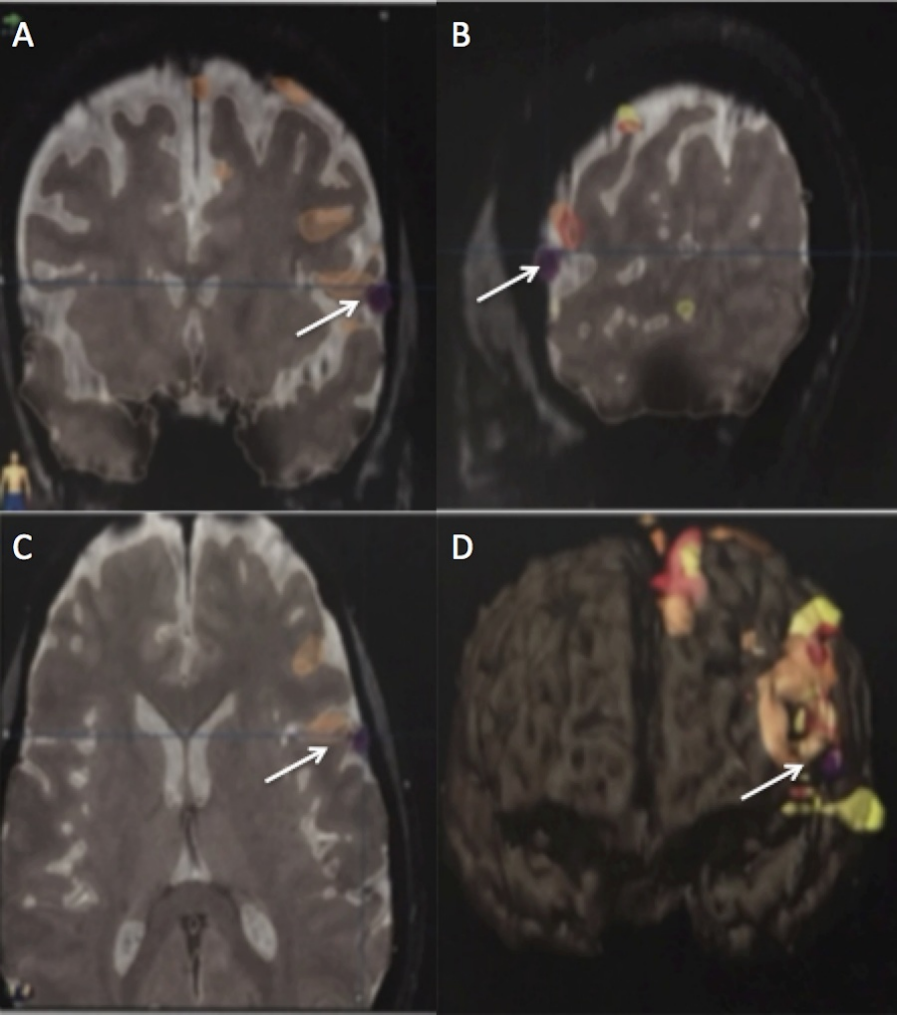
Preoperative fMRI of patient-one showing the meningioma to be marginating along Broca’s area White arrows point to activity in Broca's area

A 48-year-old right-handed female presented in May 2015 with severe weakness, vision loss, and slurred speech. Her spells were accompanied by dysphasia and a sense that her tongue was large or heavy. She had recurring episodes of migraine headaches and episodes of tunnel vision and anxiety, although it was not clear if these were secondary to anti-epileptic drugs (AEDs). Electroencephalogram (EEG) testing, done twice, showed no seizure activity despite the seizure-like episodes she described. Her magnetic resonance imaging (MRI) revealed a left frontal calcified extra-axial lesion superior to the Sylvian fissure measuring 5 mm x 8 mm x 8 mm (Figure 1). The patient insisted that her speech was affected, although people she conversed with did not notice the change. A workup was done to rule out a transient ischemic attack (TIA). The patient subsequently underwent functional magnetic resonance imaging (fMRI) of the brain to investigate the tumor’s relationship to the symptoms. The fMRI was done with axial Epibold, three dimensional (3D) fast spoiled gradient echo (FSPGR) brain volume imaging, 3D sagittal T2, susceptibility-weighted imaging (SWI), and diffuse tensor imaging (DTI) sequences. Four functional paradigms were administered by the attending neuroradiologist (Table 1). The fMRI showed that the patient was left hemisphere dominant and that the lesion directly marginated Broca’s area as revealed by the language paradigms (Figure 2). This lesion was immediately adjacent to the pars triangularis of the left inferior frontal lobe and directly marginated Broca’s area on all three language paradigms. Eloquent language cortex surrounded this lesion that was in direct contact with Broca’s area.

**Table 1:** Functional Magnetic Resonance Imaging Paradigms

Functional Paradigm	Student T-test value (p<.00001)
Motor-Bilateral finger tapping	10.0
Language-Letters to word generation from consonants	10.0
Language-Verb generation	8.0
Language-Sentence completion	8.0

**Figure 3:**
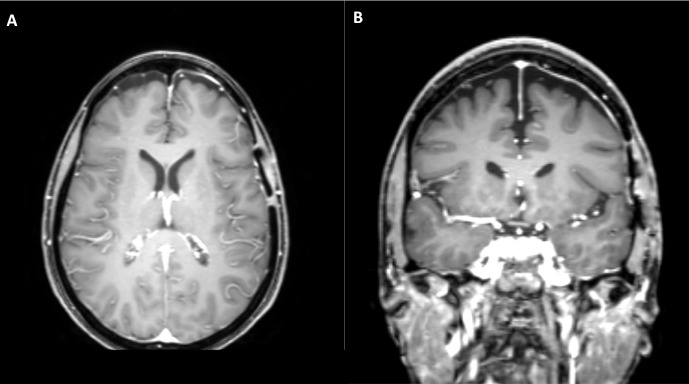
Postoperative magnetic resonance imaging of patient one A) Axial and B) coronal post contrast T1 images demonstrate gross total resection of the meningioma

**Figure 4:**
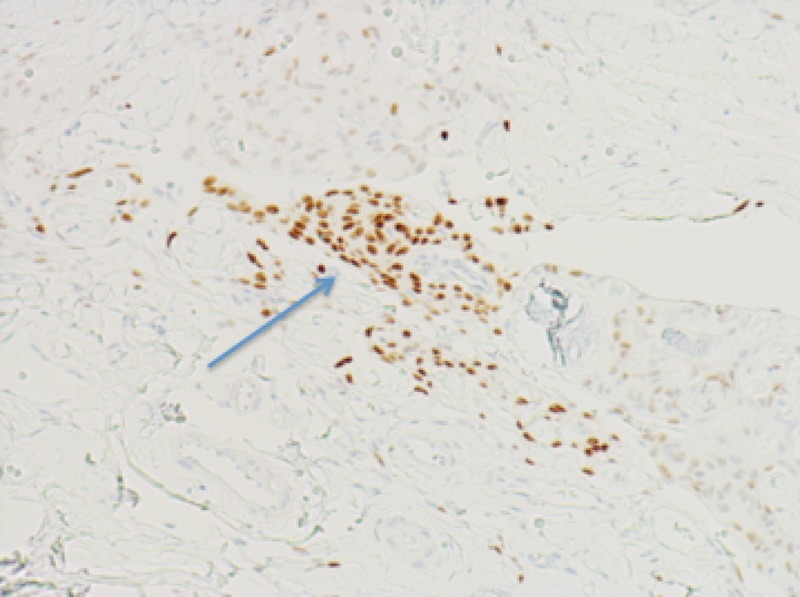
Immunohistochemistry showing minimal progesterone receptor expression for patient one Blue arrow shows minimal progesterone receptor expression

The patient underwent a gross total resection (GTR) of the tumor in September 2016 (Figure 3). Pathology showed a World Health Organization (WHO) Grade I meningioma, with Ki67 approximately 2%. Immunohistochemistry identified very few progesterone receptors (Figure 4). At her seven-month postoperative visit, the MRI showed no residual or recurrent meningioma and the patient no longer experienced language symptoms. Her neurologist was continuing a slow taper off AEDs.

Case 2

**Figure 5:**
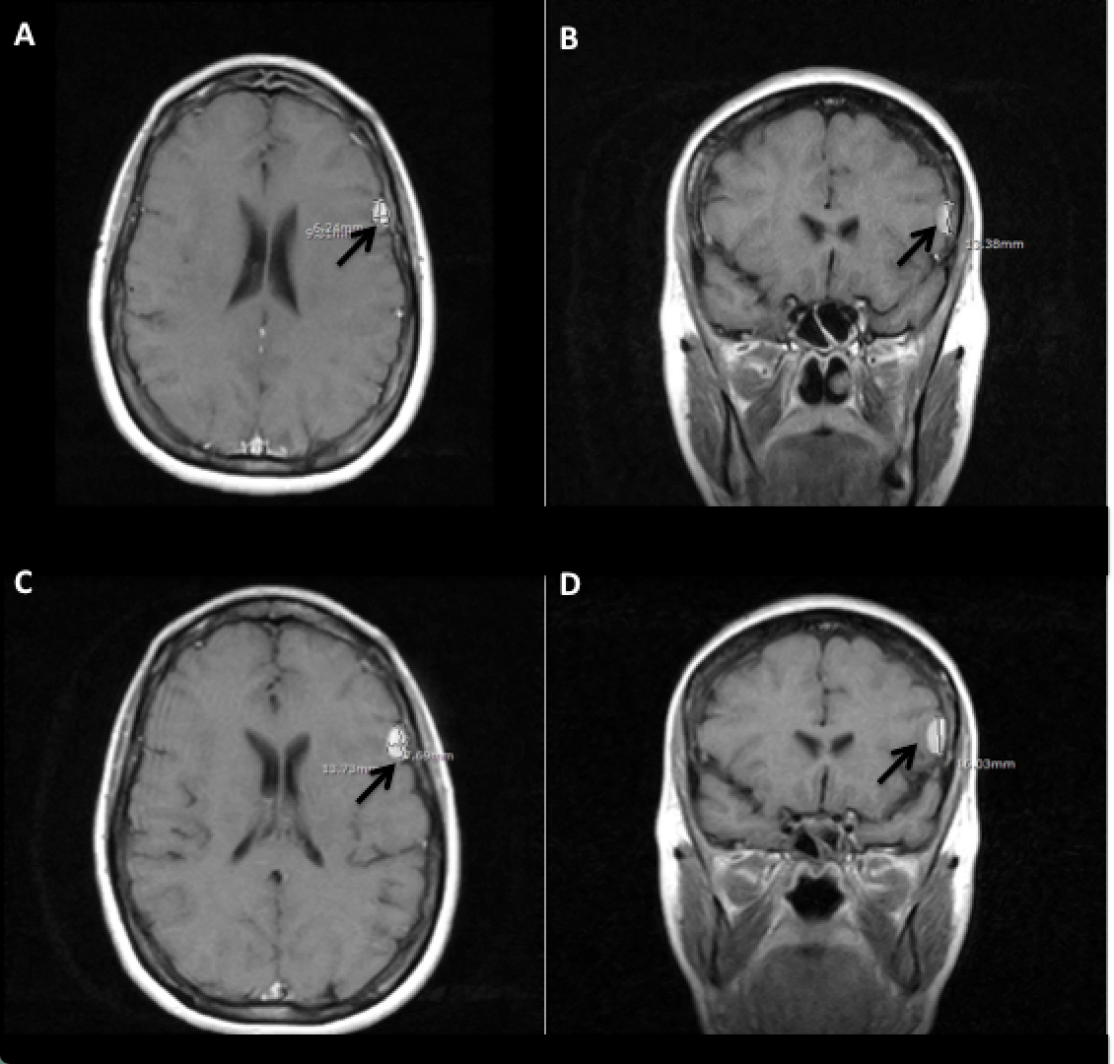
Magnetic resonance imaging illustrates meningioma growth over one year Black arrows point to lesion. (A) Axial and (B) Coronal magnetic resonance imaging show meningioma from July 2015 at 6.2 mm x 9.8 mm x 13.4 mm; (C) Axial and (D) Coronal magnetic resonance imaging show meningioma from June 2016 at 7.7 mm x 13.7 mm x 16.0 mm

A 36-year-old right-handed female presented with vertigo and left-sided pulsatile headaches in November 2015. The patient’s neurological exam did not reveal any abnormalities. MRI of the brain showed a left frontal 6.2 mm x 9.8 mm x 13.4 mm lesion (Figure 5A-B). The attending neurosurgeon recommended close observation and follow-up in six months given the tumor’s location in the dominant speech and language areas. In June 2016, the MRI showed a slight increase in the size of the meningioma to 7.7 mm x 13.7 mm x 16.0 mm and the neurosurgeon encouraged resection, given her young age (Figure 5C-D). The patient’s headaches had persisted, although she denied any other neurological symptoms.

**Figure 6:**
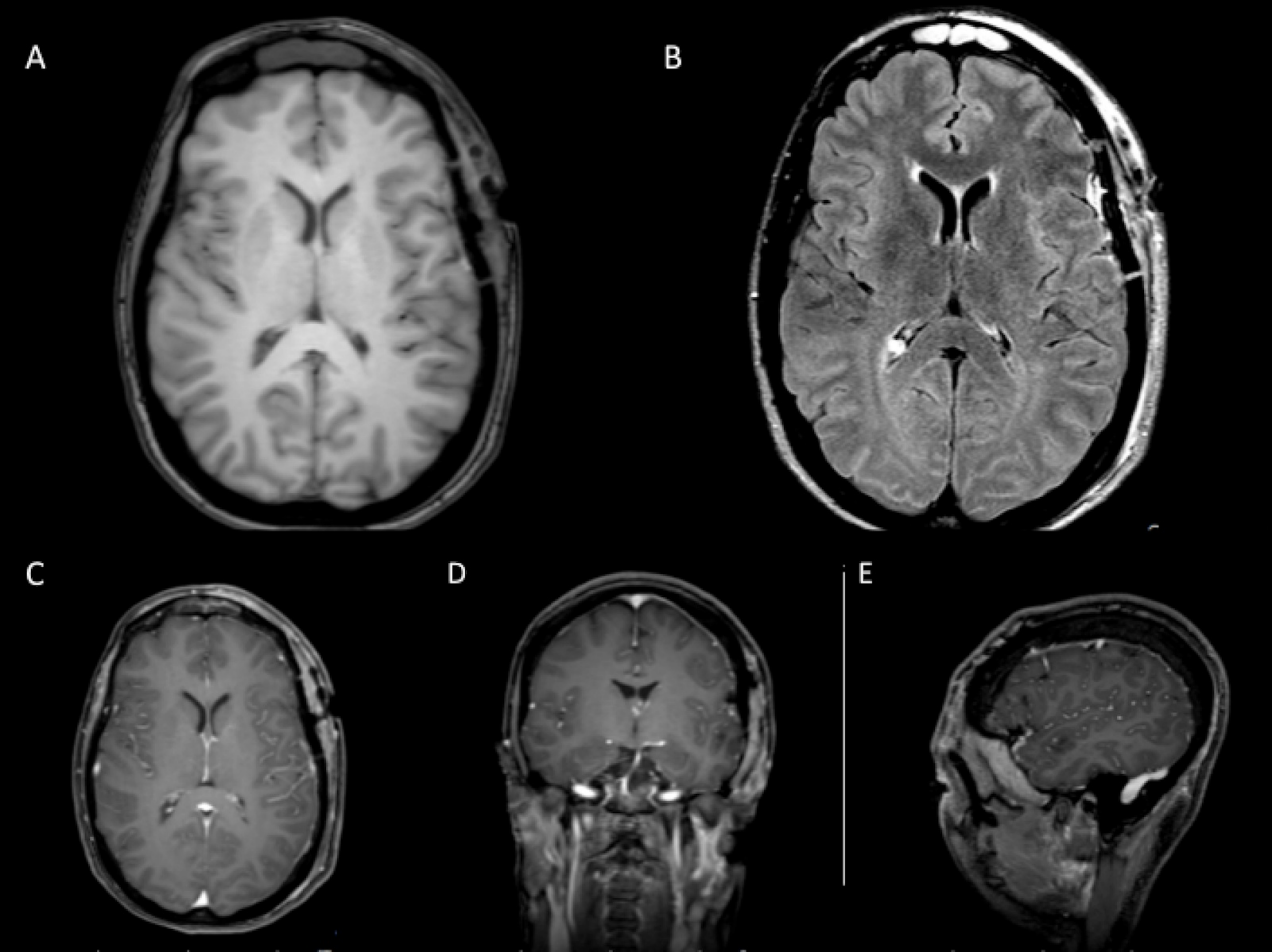
Postoperative imaging for patient two showing gross total resection Magnetic resonance imaging A) non-contrast axial T1, B) axial fluid-attenuated inversion recovery, and April 2017 follow-up C) axial, D) coronal, and E) sagittal T1

**Figure 7:**
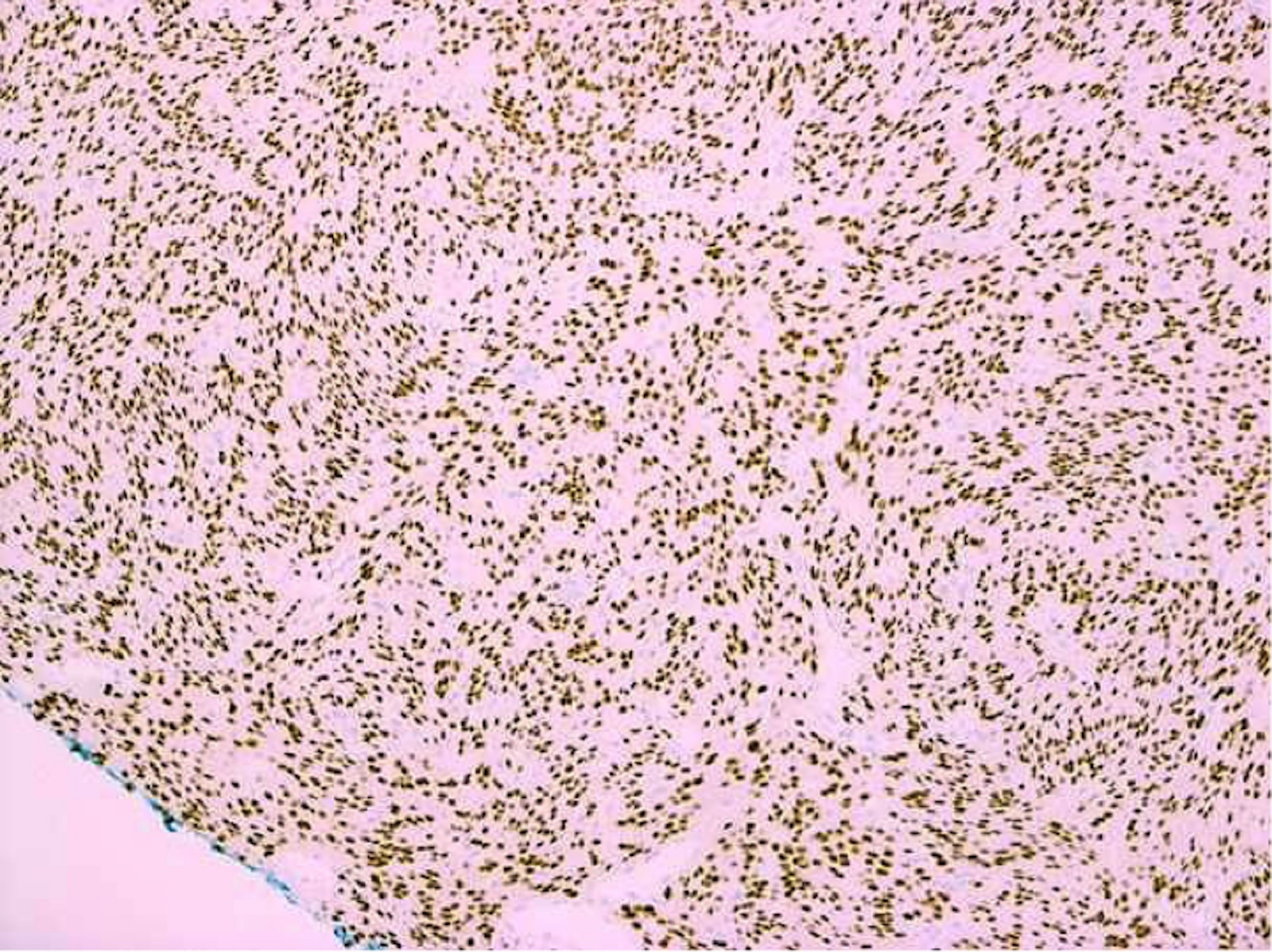
Immunohistochemistry demonstrating high levels of progesterone receptor expression for patient two

The patient underwent GTR of the tumor in September 2016 (Figure 6A-B). Pathology confirmed a WHO Grade I meningioma and immunohistochemistry showed the tumor cells to be strongly and diffusely positive for progesterone receptor (PR) expression (Figure 7). The patient reported to have received progesterone shots in November 2014 during her pregnancy. At her seven-month postoperative visit, the MRI showed no residual or recurrent meningioma and the patient’s headaches had resolved (Figure 6C-E).

## Discussion

In this case series, we illustrate the role of surgical resection of two very small left frontal meningiomas in two right-handed female patients. The first patient had a calcified meningioma, suggesting that the tumor would grow slowly, if at all [6]. In a study done by Yano et al. examining 1,434 cases of meningiomas, investigators observed that significant calcification was apparent in lesions in which there was no observed tumor growth [8]. Similarly, the first patient’s tumor did not grow; however, it was causing her speech changes and seizures. The patient reported subtle, slurred speech during which she could not verbally articulate her thoughts. Chaichana et al., retrospectively reviewed 626 patients who were diagnosed with a meningioma, of which 13% presented with seizures [5]. Although the authors concluded that larger tumors were more likely to cause seizures, our first patient presented with seizure-like symptoms that impaired her language.

The second patient’s meningioma grew during a six-month period. Given the tumor’s location in the speech and language areas and her young age, the neurosurgeon advised resection. Interestingly, the meningioma of this patient stained positively for progesterone receptors. There has been speculation about a relationship between meningiomas and hormone receptors due to the increasingly higher incidence of these tumors in the female population [2, 9-10]. This patient, whose meningioma slightly increased in size, reported having received progesterone shots during her pregnancy the year before the tumor was initially found. It has been suggested that during pregnancy, when levels of circulating progestins are high, meningiomas tend to enlarge, but nonetheless, are associated with a positive prognosis [9]. Hsu et al. found that progesterone receptor status was significantly correlated with meningioma WHO grade and a higher proportion of malignant tumors were found to have negative progesterone receptor expression [9]. Our patient had a WHO Grade I meningioma and positive progesterone receptor expression. Blitshteyn et al. conducted a retrospective study in which there was a positive association between a diagnosis of meningioma and hormone replacement therapy [10]. Investigators reported the prevalence of meningiomas in women with current or past hormone replacement therapy (HRT) to be 865 in 100,000 versus women without a history of HRT to be 366 in 100,000 [10]. However, there still needs to be more research done to assess the causality between hormone treatments and the growth of meningiomas. In both cases, GTR of each small meningioma in this eloquent location was indicated.

Despite the treatment plan for tiny meningiomas being vague, stereotactic radiosurgery (SRS) has been established as an option for patients who decline surgical resection. SRS has shown to have more favorable side effects and a 90% tumor control rate for small meningiomas [7]. Thus, patients with small meningiomas who are symptomatic or asymptomatic should be counseled to consider SRS, if appropriate. In the first case, surgery was recommended due to the lesion causing seizures and speech impairment. In the second case, the tumor grew. Both lesions were in Broca's area. Neurological symptoms and/or growth are indications for the meningioma to be removed, regardless of size, and even very small meningiomas need to be monitored. 

## Conclusions

This report of two patients with two very small meningiomas, in an eloquent area, demonstrates that small meningiomas should be considered for surgical resection when symptomatic and/or growing. Although not initially apparent, small meningiomas may be the source of seizure-like symptoms. Hormone receptors on meningiomas should not be overlooked as further investigation may show progesterone’s role in meningioma growth and WHO grade. 
